# The Postoperative Effects of Patellar Eversion in Total Knee Arthroplasty: An Updated Systematic Review and Meta-Analysis

**DOI:** 10.1155/2022/2454337

**Published:** 2022-04-28

**Authors:** Jun Wang, Jian-Bin Guo, Peng-Fei Wen, Yu-Min Zhang, Wei Song, Tao Wang, Tao Ma, Qian-Yue Cheng, Bin-Fei Zhang

**Affiliations:** Department of Joint Surgery, Honghui Hospital, Xi'an Jiaotong University, Beilin District, Xi'an, Shaanxi Province, China

## Abstract

**Background:**

During total knee arthroplasty (TKA), surgeons mobilize the patella to facilitate clear visualization of the articular surfaces and allow better prosthesis placement. According to the manipulation, this manipulation can be divided into patellar eversion and noneversion. However, the effect of patellar eversion in TKA is controversial, with substantial variability in clinical practice. This systematic review is aimed at assessing the adverse effects of patellar eversion and patellar noneversion duration in TKA.

**Methods:**

This updated systematic literature review identified randomized controlled trials comparing patellar eversion and noneversion durations in TKA. Two investigators independently extracted data and evaluated the quality of the studies. A meta-analysis was performed using RevMan version 5.3.

**Results:**

Nine studies with a total of 608 patients (730 knees) were included. Of these, 374 knees were classified in the eversion group and 356 knees in the noneversion group. The quality of the studies was high. The results showed that patellar eversion could increase the postoperative complication rate (relative risk [RR] = 1.67; 95% confidence interval [CI], 1.09–2.54; *P* = 0.02) and postoperative pain before discharge (mean deviation [MD] = 0.19; 95% CI, 0.04–0.34; *P* = 0.01), compared to noneversion. Additionally, patellar eversion could prolong the time until the patient is able to raise the leg while straightened (MD = 0.42; 95% CI, 0.24–0.59; *P* < 0.00001) and increase the length of stay (MD = 0.65; 95% CI, 0.05–1.25; *P* = 0.03). However, patellar eversion did not influence postoperative pain at 1 year (MD = 0.02; 95% CI, -0.23–0.28; *P* = 0.85), operative time (MD = −2.66; 95% CI, -8.84–3.52; *P* = 0.40), recovery of quadriceps force throughout the follow-up period, and Insall–Salvati ratio (MD = −0.04; 95% CI, [-0.11–0.02]; *P* = 0.23).

**Conclusions:**

The patellar eversion could increase the postoperative complication rate and postoperative pain. Current evidence supports the avoidance of patellar eversion in TKA. Further large-sample and long-term trials are required to validate these results.

## 1. Introduction

Total knee arthroplasty (TKA) has become one of the most vital operative strategies for patients with severe knee arthritis [1]. This technique provides excellent outcomes for deformity correction, pain relief, and functional improvement [2, 3]. During the operation, surgeons mobilize the patella to facilitate clear visualization of the articular surfaces and allow better prosthesis placement. This mobilization can be of two types based on the manipulation: patellar eversion and noneversion.

In patellar eversion, the patella is twisted along the longitudinal axis of the quadriceps mechanism and laterally retracted [4]. Eversion has the advantage of better exposure but results in perioperative torsion and increased tension on the extensor mechanism. This insult may potentially cause fibrosis and scarring of the quadriceps and patellar tendons, which may lead to patellar baja and decreased mechanical advantage of the extensor mechanism with a less optimal position of the patella in the flexion and extension arc [5–7]. Patellar noneversion requires retraction or subluxation of the patella without eversion and is increasingly performed by orthopedic surgeons. Lateral retraction or subluxation of the patella provides suboptimal surgical exposure. This can potentially increase the risk of component malposition, lateral tibial overhang, and traction damage to the patellar tendon [8].

Several randomized controlled trials (RCTs) have been conducted 2007 to 2021 to compare the relative effects of patellar eversion and patellar noneversion during TKA. These individual RCTs involved a small sample size, making the results inconclusive. Additionally, three classical meta-analyses [9–11] and one overlapping meta-analysis [12] from 2015 to 2019 compared the two types of surgical exposure. However, the conclusions of these studies are inconsistent. The reason for this difference is that the primary or interesting endpoints in these reviews varied. Zan et al. [11] and Jia et al. [9] concluded that patellar eversion and lateral patellar retraction achieved similar clinical outcomes. In contrast, Yang et al. [10] concluded that patellar noneversion leads to a shorter hospital stay and lower incidence of postoperative complications in the same RCTs. Several new RCTs have reported on this topic.

In this updated systematic review, we mainly compared two adverse postoperative effects: complications and pain. We hypothesize that patellar eversion contributes to more complications and serious pain. Therefore, this meta-analysis will determine the adverse effects of patellar eversion and patellar noneversion based on the duration of TKA and compare the results.

## 2. Materials and Methods

The study was performed according to the Preferred Reporting Items for Systematic Reviews and Meta-Analyses (PRISMA) [13] and Assessing the Methodological Quality of Systematic Reviews (AMSTAR) guidelines. We have registered the study as INPLASY PROTOCOL [14].

### 2.1. Inclusion and Exclusion Criteria

The inclusion criteria were based on the PICOS principle: (1) population: patients of all ages and sexes who required primary TKA; (2) intervention: patellar eversion duration in TKA; (3) comparison: patients who did not undergo patellar eversion during the procedure; (4) outcomes: postoperative complications, postoperative pain, operative time, length of hospitalization, quadriceps strength, straightened leg raising (SLR), and Insall–Salvati ratio follow-up period; (5) study design: RCTs. The exclusion criteria were case series studies without comparison groups and those that did not report the outcomes of interest.

### 2.2. Literature Search

We searched the studies in MEDLINE, Embase, and Cochrane Library databases. The searching strategy was (TKA OR total knee arthroplasty OR TKR OR total knee replacement) AND (eversion) AND (patellar). The retrieval dates included the period from database creation to August 2021. There were no limitations to the search process.

### 2.3. Outcome Measures

The primary endpoints were postoperative complications and pain. The secondary endpoints included operative time, length of hospitalization, quadriceps strength, SLR, and Insall–Salvati ratio follow-up period. The complications included revision, rupture or avulsion of the patellar tendon, wound infection, patella baja or tilt, and pulmonary embolism. We used a visual analog scale (VAS) to measure postoperative pain intensity.

### 2.4. Data Extraction and Quality Evaluation

We screened all titles of the retrieved articles and removed duplicates. After eliminating irrelevant articles, the summaries of the remaining articles were assessed to confirm the adequacy of the information, followed by full texts reading. Two investigators resolved disagreements through discussion, and unresolved disagreements were discussed with a third investigator.

The methodological quality was evaluated using the assessment tool recommended by the Cochrane Handbook for Systematic Reviews of Interventions [15]. The authors independently assessed each included study. Disagreements were resolved by discussion and, if necessary, scrutiny by a third reviewer. For each study, the risk of bias was categorized as low, high, or unclear.

### 2.5. Statistical Methods

RevMan version 5.3 (The Cochrane Collaboration, Copenhagen, Denmark) was used to perform analyses. Relative risk (RR) and weighted mean differences (WMDs) were used as effect sizes, with 95% confidence intervals (CIs). The statistical methods included the Mantel–Haenszel and inverse variance tests. Heterogeneity was assessed using the *I*^2^ statistic. A fixed-effects model was employed during quantitative synthesis for low heterogeneity (*I*^2^ < 50%, *P* > 0.1). When heterogeneity was high (*I*^2^ > 50%, *P* < 0.1), we first explored the possible sources of heterogeneity or used a random effects model. *P* < 0.05 was considered statistically significant at *P* < 0.05.

## 3. Results

### 3.1. Included Studies

**Figure 1:**
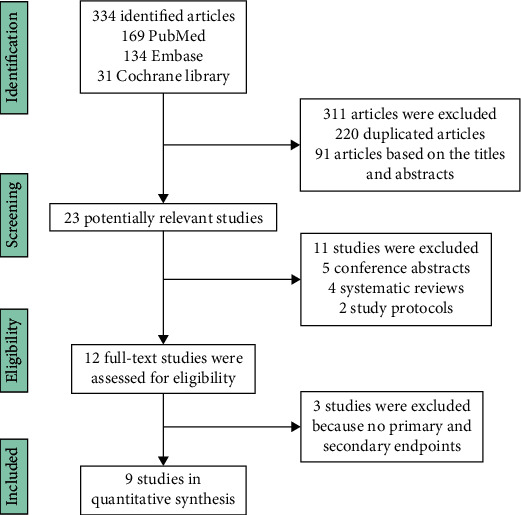
Flowchart of the studies included in the meta-analysis.

After the search, 334 eligible articles were obtained. However, most were excluded because of duplicates and lack of relevance. After screening and assessment, nine studies corresponded to the inclusion criteria. The selected studies were written in English and published between 2009 and 2021.  Figure 1 shows the flow of the study throughout the trial.

### 3.2. Characteristics and Quality Evaluation of the Included Studies

Nine studies with 608 patients (730 knees) were included. The 85 patients in three RCTs [16–18] received simultaneous bilateral TKAs, while those in the remaining seven studies [19–24] received unilateral TKA. Of these, 374 knees were classified in the eversion group and 356 in the noneversion group. The sample size ranged from 61 [23] to 120 [20] patients. Seven studies [16–20, 22, 24] reported that patients were diagnosed with osteoarthritis, and the remaining two studies [21, 23] did not report the diagnosis. All RCTs reported the surgical approach: medial parapatellar approach in seven studies [16–21, 24] and midvastus approach in two studies [22, 23]. Regarding the patella, eight studies [16, 17, 19–24] reported the procedure: resurfacing in four studies [19, 20, 22, 23] and no resurfacing in four studies [16, 17, 21, 24]. The follow-up duration varied from 3 months to 1 year (Table 1).

Table 2 showed the summarized results of each RCT including number and kind of complications, pain, hospital stay, and operation time.

**Figure 2:**
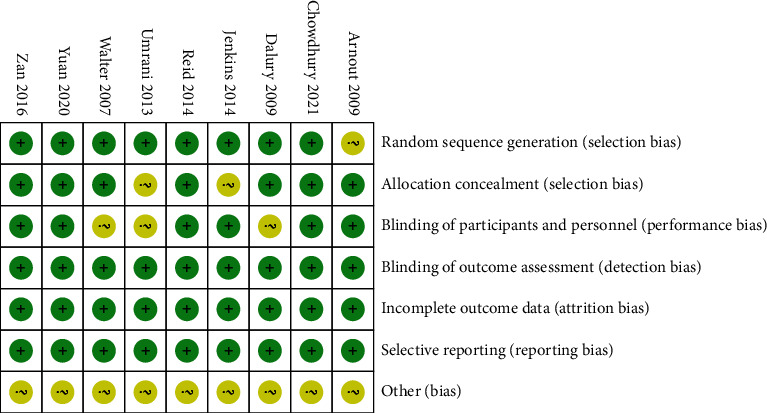
Risk of bias summary.

**Figure 3:**
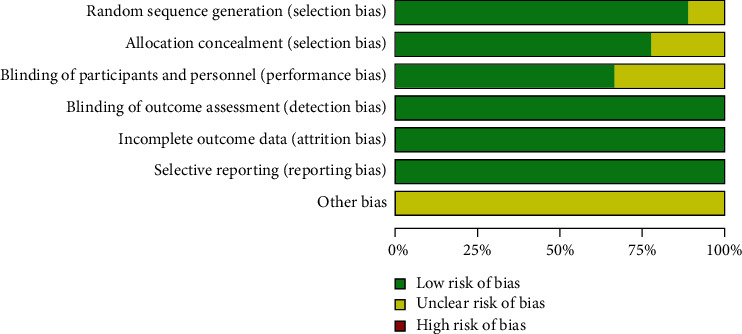
Risk of bias graph.

The quality of the included studies was assessed according to the Cochrane Handbook for Systematic Reviews of Interventions (Figures 2 and 3). Among the nine RCTs, eight studies [16–18, 20–24] described adequate methods of random sequence generation; the method of randomization in Arnout et al. [19] was not mentioned. Allocation concealment was well described in seven trials [16–19, 21, 23, 24] and unclear in the other two studies [20, 22]. However, patients were blinded to the procedure in six studies [16, 17, 19–21, 24], and three studies did not report detailed information on blinding of the participants [18, 22, 23]. Additionally, all included studies reported blinding of the outcome assessors.

### 3.3. Primary Endpoints

#### 3.3.1. Postoperative Complications

**Figure 4:**
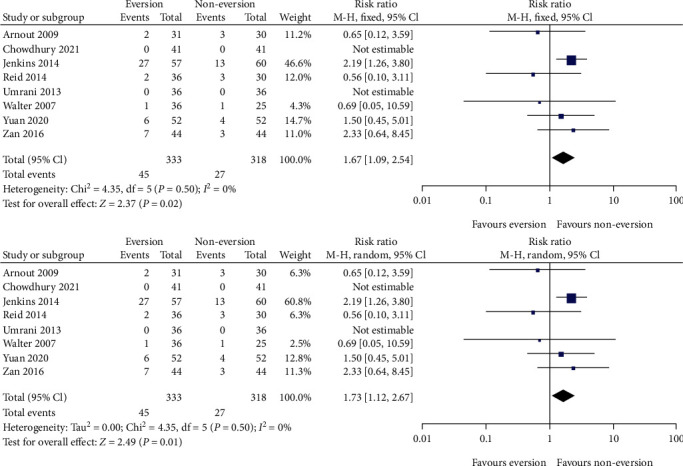
Forest plot comparing complications between the patellar eversion and noneversion groups (fixed-effects model and random-effects model).

As shown in Figure 4, eight studies [16, 17, 19–24] reported complications. The *I*^2^ value for heterogeneity was 0% (*P* = 0.50); therefore, a fixed-effects model was applied. Postoperative complications in the eversion group were higher than those in the noneversion group (RR = 1.67; 95% CI, 1.09–2.54; *P* = 0.02). When the aggregate results of these studies were changed to a random-effects model, the result was the same as that of the fixed-effects model (RR = 1.73; 95% CI, 1.12–2.67; *P* = 0.01). When we introduced subgroups according to the surgical approach, the results from the midvastus approach showed no significant differences between the eversion and noneversion groups (RR = 0.69; 95% CI, 0.05–10.59; *P* = 0.79). The results from the medial parapatellar approach showed that postoperative complications in the eversion group were higher than those in the noneversion group (RR = 1.71; 95% CI, 1.11–2.63; *P* = 0.01).

After we introduced subgroups according to the presence or absence of patellar resurfacing, we found that postoperative complications in the eversion group were higher than those in the noneversion group with resurfacing (RR = 1.80; 95% CI, 1.09–2.99; *P* = 0.02). The results without resurfacing showed no statistical difference (RR = 1.44; 95% CI, 0.68–3.08; *P* = 0.34).

#### 3.3.2. Pain

**Figure 5:**
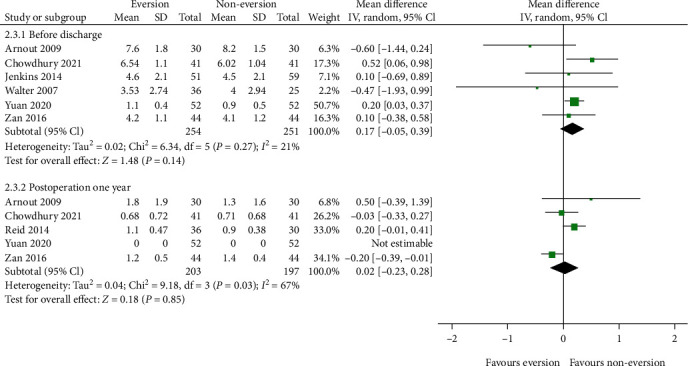
Forest plot comparing pain between the patellar eversion and noneversion groups.

We introduced two subgroups to assess pain: before discharge and 1 year postoperatively. Six studies [16, 17, 19, 20, 23, 24] reported pain before discharge. The aggregate resulted in an *I*^2^ value for heterogeneity of 21% (*P* = 0.27); thus, a fixed-effects model was used. The eversion group had higher pain intensity than the noneversion group (MD = 0.19; 95% CI, 0.04–0.34; *P* = 0.01). However, the results were not stable when the model was changed to a random-effects model (Figure 5). Additionally, five studies [16, 17, 19, 21, 24] reported pain at 1 year postoperatively. Using the random-effects model, we found no statistical difference between the two groups (MD = 0.02; 95% CI, -0.23–0.28; *P* = 0.85).

### 3.4. Secondary Endpoints

#### 3.4.1. Operative Time

**Figure 6:**
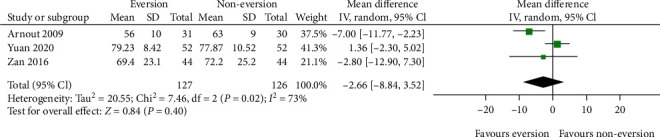
Forest plot comparing operation time between the patellar eversion and noneversion groups.

Three studies [17, 19, 24] reported the operative time. As shown in Figure 6, the aggregate resulted in an *I*^2^ value for heterogeneity of 73% (*P* = 0.02); thus, the random-effects model was used. There was no significant difference in the operative time between groups (MD = −2.66; 95% CI, -8.84–3.52; *P* = 0.40).

#### 3.4.2. Length of Hospital Stay

**Figure 7:**
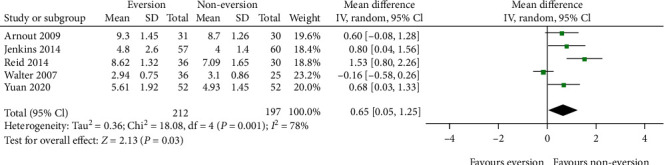
Forest plot comparing length of stay between the patellar eversion and noneversion groups.

As shown in Figure 7, five studies [19–21, 23, 24] reported the length of hospital stay. As the *I*^2^ value for heterogeneity was 78% (*P* = 0.001), the random-effects model was used. The eversion group had a significantly longer stay than the noneversion group (WMD = 0.65; 95% CI, 0.05–1.25; *P* = 0.03). However, the results did not show stability in the sensitivity analysis when individual studies were excluded.

#### 3.4.3. Quadriceps Function

Quadriceps function included quadriceps strength and time to return of SLR. Four studies [18–20, 22] measured quadriceps strength using a dynamometer. Umrani et al. [22], Arnout et al. [19], Dalury et al. [18], and Jenkins et al. [20] reported that there were no statistical differences between the two groups throughout the follow-up period in the recovery of quadriceps force. Three studies [17, 23, 24] reported the time to return to SLR. The aggregate resulted in an *I*^2^ value for heterogeneity of 1% (*P* = 0.36); thus, the fixed-effects model was used. We found that the time to return of SLR in the eversion group was longer than that in the noneversion group (MD = 0.42; 95% CI, 0.24–0.59; *P* < 0.00001). This result was stable when the random-effects model was used.

#### 3.4.4. Insall–Salvati Ratio

Four studies [19–21, 24] reported the Insall–Salvati ratio. The aggregate resulted in an *I*^2^ value for heterogeneity of 88% (*P* < 0.001); thus, the random-effects model was used. We found no significant difference in the Insall–Salvati ratio between the two groups (MD = −0.04; 95% CI, [-0.11–0.02]; *P* = 0.23).

## 4. Discussion

In this systematic review, we added three new studies [16, 17, 24] and updated the clinical evidence regarding the effects of patellar eversion in TKA. There were 189 patients in these three studies, including 137 knees that underwent patellar eversion and 137 knees that underwent patellar noneversion. Overall, the results showed that patellar eversion could increase the postoperative complication rate and postoperative pain intensity before discharge, as compared to the noneversion group. Additionally, patellar eversion could prolong the time for return of SLR and increase the length of hospital stay. However, patellar eversion did not influence pain at 1 year postoperatively, operative time, recovery of quadriceps force throughout the follow-up period, and the Insall–Salvati ratio. This updated meta-analysis showed different results from previous meta-analyses [9, 11] and one overlapping meta-analysis [12]. Previous published review sets the clinical outcome as primary outcome, and they found that no difference in clinical outcome between patella eversion and noneversion [9, 11, 12], while the complications and pain were set as primary outcomes in this meta-analysis because the effect of surgical management of the patella is mainly on adverse effect in early knee functional recovery [16]. In additional, there were more patients in these nine RCTs than Yang et al. [10] on this topic.

The complications included revision, rupture or avulsion of the patellar tendon, wound infection, patella baja or tilt, and pulmonary embolism. The overall complication rates were 13.5% (45/333) and 8.5% (27/318) in the eversion and noneversion groups, respectively. As for TKA revision, there were no reported cases in the short term. Regarding rupture or avulsion of the patellar tendon, pooled results from six studies [18–23] did not show a difference between the two groups (RR = 1.12; 95% CI, 0.29–4.36; *P* = 0.87). Regarding patella baja or tilt, pooled results from five studies [11, 16, 20, 21, 24] did not show a difference between the two groups (RR = 0.99; 95% CI, 0.31–3.15; *P* = 0.99).

Regarding the overall complications, when introducing subgroups according to the surgical approach, we found that the medial parapatellar approach had a postoperative complication rate compared to the midvastus approach. In a retrospective analysis of 875,166 elective operations, Blom et al. [25] reported that the midvastus approach was associated with lower revision rates than the medial parapatellar approach. In a meta-analysis, Alcelik et al. [26] found that the midvastus approach did not increase the complication rates. After introducing subgroups according to patellar resurfacing, we found that TKA with resurfacing was associated with higher postoperative complications compared to those without resurfacing. In a prospective RCT, Deroche et al. [27] reported that patellar tilt occurred in 43% of resurfaced knees and 29% of nonresurfaced knees. Crawford et al. [28] reported that patients with patellar resurfacing had a significantly higher incidence of manipulation under anesthesia than those without resurfacing. Choe et al. [29] reported that complications after TKA without patellar resurfacing are infrequent.

Regarding postoperative pain, we divided the results into before discharge and at 1 year postoperatively in our analysis. We found that patellar eversion increased the postoperative pain before discharge. The duration of the operation and traction force to the muscle by patellar eversion may affect the recovery of muscle strength and increase pain [30]. Majima et al. [30] found that the noneversion group showed a lower VAS score than the eversion group until 4 weeks postoperatively. The reason the previous meta-analysis drew contradictory conclusions might be the short subgroups of time points. Moreover, the inconsistent use of tourniquets may lead to the development of different levels of lower limb ischemia-reperfusion injury, which might affect the evaluation of anterior knee pain. Another reason might be that knee pain was measured using VAS, which is a subjective scale scored by the patients themselves [16]. However, postoperative pain at 1 year postoperatively was comparable between the two groups. We considered that the side effects of patellar eversion would decrease.

Regarding the secondary endpoints, patellar eversion could prolong the time for return of SLR and increase the length of hospital stay but did not influence the operative time, recovery of quadriceps force, or the Insall–Salvati ratio. These endpoints reflected functional recovery. Generally, patellar eversion during surgery could delay short-term postoperative recovery but not long-term functional recovery.

Based on these results, we recommend the avoidance of patellar eversion in clinical practice. However, the surgeons' habits and knee anatomy in patients vary greatly. If the surgeon can implant the component in a good position without tibial overhang, there is no need to reverse the patella. The quality of RCTs is an important factor. In the included RCTs, most studies reported vital elements according to guidance. The risk of bias was relatively low.

This systematic review had some limitations. First, two studies [21, 23] did not report the diagnosis and required only primary TKA for patient inclusion. We could not identify patients with rheumatoid or traumatic arthritis, which should be analyzed in the subgroups. Second, slight clinical heterogeneity was observed due to differences in the surgical approach, patellar procedure, and follow-up between studies. These factors may have contributed to the heterogeneity. Third, we tried to determine the confounding factors by metaregression, but failed because the number of included studies was small. Therefore, we could not evaluate possible confounding factors, including bone mineral density and type of prosthesis. Thus, these results should be interpreted with caution.

## 5. Conclusions

The patellar eversion could increase the postoperative complication rate and postoperative pain. Current updated evidence supports avoidance of patellar eversion in TKA. Further large-sample and long-term trials are required to validate our results.

## Figures and Tables

**Table 1:** Characteristics of the included studies.

Study	No. of patients	No. of knees		Inclusion criteria	Approach	Procedure of patella	Outcomes	Follow-up
		Eversion	No eversion					
Arnout 2009 [19]	61	31	30	Osteoarthritis	Medial parapatellar approach	Resurfacing	Complications, VAS, operative time, Insall–Salvati ratio, length of stay	1 year
Chowdhury 2021 [16]	41	41	41	Osteoarthritis	Medial parapatellar approach	Without resurfacing	VAS, time to SLR, quadriceps strength	1 year
Dalury 2009 [18]	37	37	37	Osteoarthritis	Medial parapatellar approach	NA	Complications, time to SLR, quadriceps strength	6 months
Jenkins 2014 [20]	120	60	60	Osteoarthritis	Medial parapatellar approach	Resurfacing	Complications, length of stay, Insall–Salvati ratio, VAS, time to SLR	1 year
Reid 2014 [21]	68	37	31	Required primary TKA	Medial parapatellar approach	Without resurfacing	Length of stay, Insall-Salvati ratio, VAS	1 year
Umrani 2013 [22]	72	36	36	Osteoarthritis	Midvastus approach	Resurfacing	Quadriceps force	1 year
Walter 2007 [23]	61	36	25	Required primary TKA	Midvastus approach	Resurfacing	SLR, VAS, length of stay, complications	3 months
Yuan 2020 [24]	104	52	52	Osteoarthritis	Medial parapatellar approach	Without resurfacing	VAS, length of stay, complications, time to SLR	1 year
Zan 2016 [17]	44	44	44	Osteoarthritis	Medial parapatellar approach	Without resurfacing	Complications, time to SLR, VAS	1 year

**Table 2:** The summarize the results of each RCT.

Study	Complications		Pain (before discharge)		Pain (postoperation one year)		Hospital stay (days)		Operation time (mins)	
	Eversion	Noneversion	Eversion	Noneversion	Eversion	Noneversion	Eversion	Noneversion	Eversion	Noneversion
Arnout 2009 [19]	2 (1patella fracture, 1 cerebral vascular incident)	3 (1 peroneal nerve palsy, 2 wound problems)	7.6 ± 1.8	8.2 ± 1.5	1.8 ± 1.9	1.3 ± 1.6	9.3 ± 1.45	8.7 ± 1.26	56 ± 10	63 ± 9∗
Chowdhury 2021 [16]	0	0	6.54 ± 1.1	6.02 ± 1.04∗	0.68 ± 0.72	0.71 ± 0.68	—	—	—	—
Dalury 2009 [18]	—	—	—	—	—	—	—	—	—	—
Jenkins 2014 [20]	27 (7 pulmonary emboli, 2 partial avulsions of the patellar tendon, 8 stiffness, 2 painful crepitus, 2 delayed skin healing, 4 knee pain, 2 patella Baja)	13 (1 pulmonary emboli, 6 stiffness, 1 painful crepitus, 5 anterior knee pain or retropatellar pain) ∗	4.6 ± 2.1	4.5 ± 2.1	—	—	4.8 ± 2.6	4.0 ± 1.4∗	—	—
Reid 2014 [21]	2 (injuries to the patellar tendon)	3 (2 injuries to the patellar tendon, 1 baja)	—	—	1.1 ± 0.47	0.9 ± 0.38	8.62 ± 1.32	7.09 ± 1.65∗		
Umrani 2013 [22]	0	0	—	—	—	—	—	—	—	—
Walter 2007 [23]	1 (patellar tendon rupture)	1 (infection)	3.53 ± 2.74	4.0 ± 2.94	—	—	2.94 ± 0.75	3.1 ± 0.86	—	—
Yuan 2020 [24]	6 (1 patellar baja, 1 patellar tendon tearing, 2 incision fat liquefaction, 2 knee hematoma)	4 (2 incision fat liquefaction, 2 superficial wound infection)	1.1 ± 0.4	0.9 ± 0.5	0	0	5.61 ± 1.92	4.93 ± 1.45∗	79.23 ± 8.42	77.87 ± 10.52
Zan 2016 [17]	7 (1 tearing of the patella tendon, 3 knee hematoma, 1 deep vein thrombosis, 1 wound infection, 1 patella baja)	3 (1 knee stiffness, 1 deep vein thrombosis, 1 wound infection)	4.2 ± 1.1	4.1 ± 1.2	1.2 ± 0.5	1.4 ± 0.4	—	—	69.4 ± 23.1	72.2 ± 25.2

∗There was a statistic difference between eversion and noneversion groups.

## Data Availability

Data sharing is not applicable to this article as no datasets were generated or analyzed during the current study.

## Abbreviations

TKA: Total knee arthroplasty
SLR: Straightened leg raising
RCT: Randomized controlled trials
WMD: Weighted mean difference
CI: Confidence interval
RR: Relative risk.

## Additional Points

*Provenance and Peer Review.* It is not commissioned and externally peer-reviewed.
